# Ultrasound-Guided Caudal Epidural Block Versus Saddle Block for Anorectal Surgeries: A Prospective Randomized Comparative Study

**DOI:** 10.7759/cureus.108220

**Published:** 2026-05-04

**Authors:** Iniya Rajendran, Prasanna Vadhanan, Meenu R Ampalaya, Preethi Priyadharshini

**Affiliations:** 1 Department of Anesthesiology, Vinayaka Mission's Medical College, Karaikal, IND

**Keywords:** anorectal surgery, caudal epidural block, early mobilisation, postoperative analgesia, saddle block, ultrasound guidance, ultrasound-guided caudal epidural block

## Abstract

Background

Anorectal surgeries are commonly performed as ambulatory procedures requiring effective perineal anesthesia, hemodynamic stability, adequate postoperative pain control, and early mobilization to facilitate same-day discharge. Saddle block provides dense sacral anesthesia but is often associated with sympathetic blockade, hypotension, motor impairment, and urinary retention. Ultrasound-guided caudal epidural block (USG-CEB) has emerged as a reliable alternative that allows selective sacral nerve root blockade with potentially improved recovery characteristics. However, direct comparative evidence between USG-CEB and saddle block in adult anorectal surgeries remains limited.

Aim and objectives

To compare USG-CEB with saddle block in adult patients undergoing elective anorectal surgery. The primary objectives were to evaluate time to mobilization and time to first rescue analgesia. Secondary objectives included assessment of onset of sensory blockade, intraoperative hemodynamic changes, time to voiding, requirement for supplemental local infiltration, and patient satisfaction.

Materials and methods

This prospective randomized controlled study included 59 adult patients scheduled for elective anorectal surgery. The trial was registered in the Clinical Trials Registry of India (CTRI/2020/01/022896) and also obtained approval from the Institutional Ethics Committee (approval number: VMMC/ANESTH/2019/01). Patients were randomized into two groups: Group C (USG-CEB) and Group S (saddle block). Ultrasound-guided blocks were performed using a high-frequency linear probe (L13-3Ns, 3-13 MHz). Rescue analgesia was administered when the visual analogue scale (VAS) score was ≥four, using intravenous tramadol 50 mg, repeatable every eight hours as required. Postoperative assessments were conducted by a blinded observer. Data were analyzed using appropriate statistical methods. Continuous variables were expressed as mean ± standard deviation and compared using the independent t-test. Categorical variables were analyzed using the chi-square test. Effect sizes (Cohen’s d) and 95% confidence intervals were calculated for primary and key secondary outcomes. A p-value <0.05 was considered statistically significant.

Results

Patients in the USG-CEB group demonstrated significantly earlier mobilization (5.82 ± 0.71 h vs. 6.95 ± 0.60 h, p<0.001) and earlier time to voiding (4.95 ± 0.73 h vs. 6.82 ± 0.71 h, p<0.001) compared to the saddle block group. Time to first rescue analgesia was longer in the USG-CEB group (5.00 ± 0.95 h vs. 4.45 ± 0.74 h, p=0.021). However, sensory onset was faster in the saddle block group (9.83 ± 1.42 min vs. 12.86 ± 1.84 min, p<0.001). Hemodynamic parameters were more stable in the USG-CEB group.

Conclusion

USG-CEB is an effective alternative to saddle block for anorectal surgery, offering a favorable recovery profile characterized by earlier mobilization, earlier voiding, and stable hemodynamics. However, these advantages should be weighed against a slower onset of anesthesia and the need for supplemental analgesia in some patients. The clinical significance of these findings may vary across practice settings, and anesthetic technique should be individualized.

## Introduction

Anorectal conditions such as hemorrhoids, fissures, fistula-in-ano, and perianal abscesses are among the most common problems encountered in surgical practice. Many of these conditions require operative management, and a large proportion of these procedures are now performed in ambulatory settings [1]. With the growing emphasis on day-care surgery, the expectations from anesthetic techniques have evolved considerably. Beyond providing adequate intraoperative anesthesia, the ideal technique should allow rapid recovery, early mobilization, minimal side effects, and timely discharge [2].

Regional anesthesia continues to be the preferred approach for anorectal procedures because of its ability to provide targeted analgesia while avoiding the systemic effects of general anesthesia. Among the available options, saddle block has long been favored due to its technical simplicity, rapid onset, and reliable sacral sensory blockade [3]. By restricting the spread of local anesthetic to the lower segments of the spinal cord, it provides effective perineal anesthesia with minimal cephalad extension. However, in clinical practice, this technique is not without limitations. Hypotension due to sympathetic blockade, urinary retention, and delayed ambulation related to motor block are well-recognized concerns [4]. These issues can be particularly relevant in ambulatory settings, where even small delays in recovery may impact discharge timing and patient turnover.

Caudal epidural block (CEB), although traditionally associated with pediatric anesthesia, has increasingly been explored as an alternative technique in adult patients undergoing anorectal surgeries. The caudal approach allows selective blockade of sacral nerve roots, thereby providing adequate perineal analgesia while potentially preserving hemodynamic stability. In theory, this more localized blockade should reduce the incidence of complications such as hypotension and urinary retention [5]. However, the uptake of CEB in adults has historically been limited by anatomical variability and the difficulty of accurately identifying the sacral hiatus using landmark-based techniques. This has often resulted in higher rates of failed or inadequate blocks.

The introduction of ultrasound guidance (USG) has significantly changed this landscape. Ultrasound allows direct visualization of key anatomical structures, including the sacral cornua and sacrococcygeal ligament, thereby improving the accuracy of needle placement. This has led to higher success rates and greater confidence among practitioners when performing CEB in adults [6]. Several studies have demonstrated that USG not only improves block reliability but may also reduce procedural complications compared to traditional blind techniques [7]. As a result, ultrasound-guided caudal epidural block (USG-CEB) has emerged as a promising option for perineal surgeries.

Despite these advancements, the relative advantages of USG-CEB compared to saddle block in adult anorectal surgery are not yet fully established. While some studies suggest that caudal techniques may offer better hemodynamic stability and improved recovery profiles, the evidence remains limited and somewhat heterogeneous [8]. Differences in study design, anesthetic regimens, and outcome measures make it difficult to draw firm conclusions. Moreover, an important question remains whether the observed benefits are due to the caudal approach itself or the addition of USG.

In the context of ambulatory surgery, outcomes such as time to mobilization, return of bladder function, and readiness for discharge are particularly important, yet these are not consistently reported in existing literature. Given these gaps, there is a clear need for well-designed comparative studies that focus specifically on recovery-related outcomes in adult patients.

The present study was undertaken with this objective in mind. We aimed to compare USG-CEB with saddle block in patients undergoing elective anorectal surgery, with particular emphasis on clinically relevant recovery parameters. We hypothesized that USG-CEB would provide comparable intraoperative anesthesia while offering a more favorable recovery profile, especially in terms of earlier mobilization and reduced postoperative complications.

## Materials and methods

Study design and ethical approval

This prospective randomized comparative study was conducted in the Department of Anesthesiology at Vinayaka Mission’s Medical College, Karaikal, India, over two years after obtaining approval from the Institutional Ethics Committee (IEC approval number: VMMC/ANESTH/2019/01). This trial was registered at Clinical Trials Registry of India (Reg. no. CTRI/2020/01/022896). The study adhered to the ethical principles outlined in the Declaration of Helsinki. Written informed consent was obtained from all participants prior to enrolment.

Sample size 

The sample size was estimated based on the primary outcome of time to mobilization. As limited prior data were available for this specific parameter, assumptions were guided by published randomized studies comparing caudal and spinal anesthesia in anorectal surgery, including the study by Chen et al. [8], along with pilot observations from our patient population. A difference of approximately one hour in mobilization time was considered clinically meaningful for ambulatory practice, with an assumed standard deviation of 0.8-1.0 hours. Using a two-sided α of 0.05 and a power of 80%, the required sample size was calculated for comparison of two independent means. To allow for potential dropouts and protocol deviations, an additional 10-15% was included, and the final sample size was adjusted accordingly to ensure adequate statistical power, which turned out to be 59.

Study population

Fifty-nine adult patients aged 18-60 years, belonging to American Society of Anesthesiologists (ASA) physical status I-III [9], scheduled for elective anorectal surgeries (hemorrhoidectomy, lateral internal sphincterotomy, fistulectomy, or perianal abscess drainage) under regional anesthesia were included. Patients with coagulopathy, local infection at the injection site, allergy to amide local anesthetics, severe cardiovascular disease, spinal deformities, raised intracranial pressure, pre-existing neurological deficits, or refusal to participate were excluded from the study.

Randomization and blinding

Allocation concealment was ensured using sequentially numbered, sealed, opaque envelopes, which were opened immediately prior to the procedure. Due to the nature of the interventions, blinding of the anesthesiologist and patients was not feasible. However, postoperative outcome assessment and data collection were performed by an investigator blinded to group allocation.

Anesthetic management

All patients were kept fasting as per standard guidelines and received no premedication. Standard monitoring including electrocardiography, non-invasive blood pressure, and pulse oximetry was instituted.

Group C: Ultrasound-guided Caudal Epidural Block (USG-CEB)

**Figure 1:**
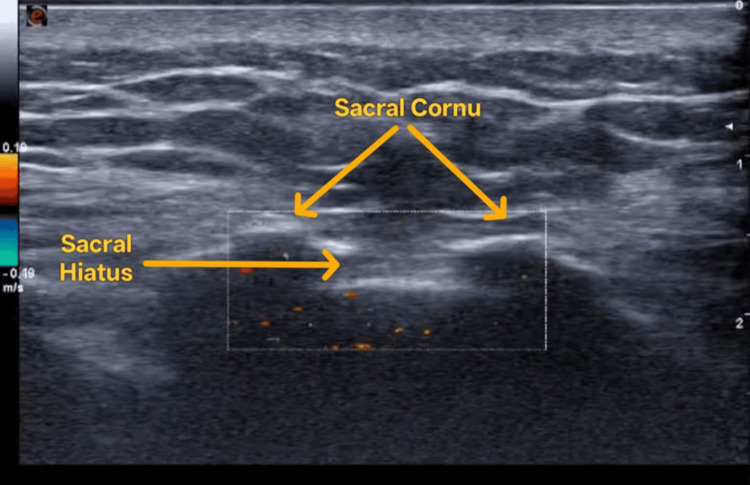
Ultrasound image showing transverse view of the sacral hiatus

Twenty-nine patients were allotted to this group. USG was performed using a Mindray ultrasound system (Mindray Medical International Limited, Shenzhen, China) equipped with a high-frequency linear transducer (L13-3Ns, frequency range 3-13 MHz). With the patient in a prone position, the sacral hiatus was palpated and the transducer was placed in the midline, the depth was adjusted between 2-4 cm to optimize visualization of the sacral hiatus, which was identified using both transverse and longitudinal views. Standard anatomical landmarks including the sacral cornua, sacrococcygeal ligament, and dorsal sacral surface were visualized. The bony prominences of the two sacral cornua appear as two hyperechoic reversed U-shaped structures. Between the two cornua, two hyperechoic band-like structures - the sacrococcygeal ligament superiorly and the dorsal bony surface of the sacrum inferiorly - can be identified, and the sacral hiatus is the hypoechoic area in between (Figure 1).

**Figure 2:**
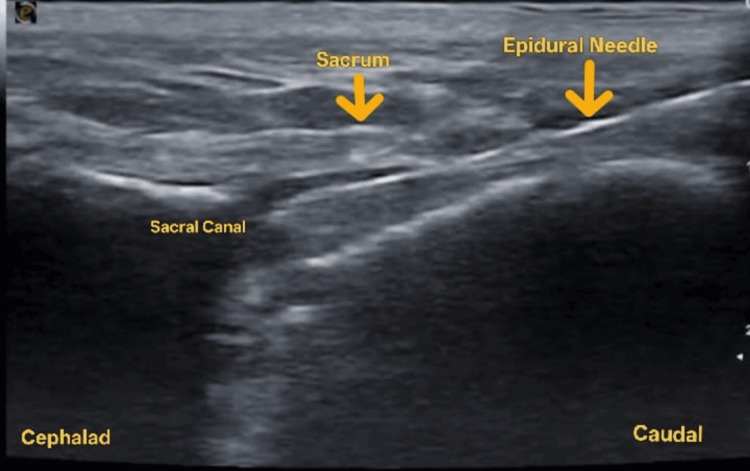
Ultrasound image showing longitudinal view of sacral hiatus

Under standard aseptic precautions, a 22G needle was inserted under ultrasound guidance (Figure 2).

**Figure 3:**
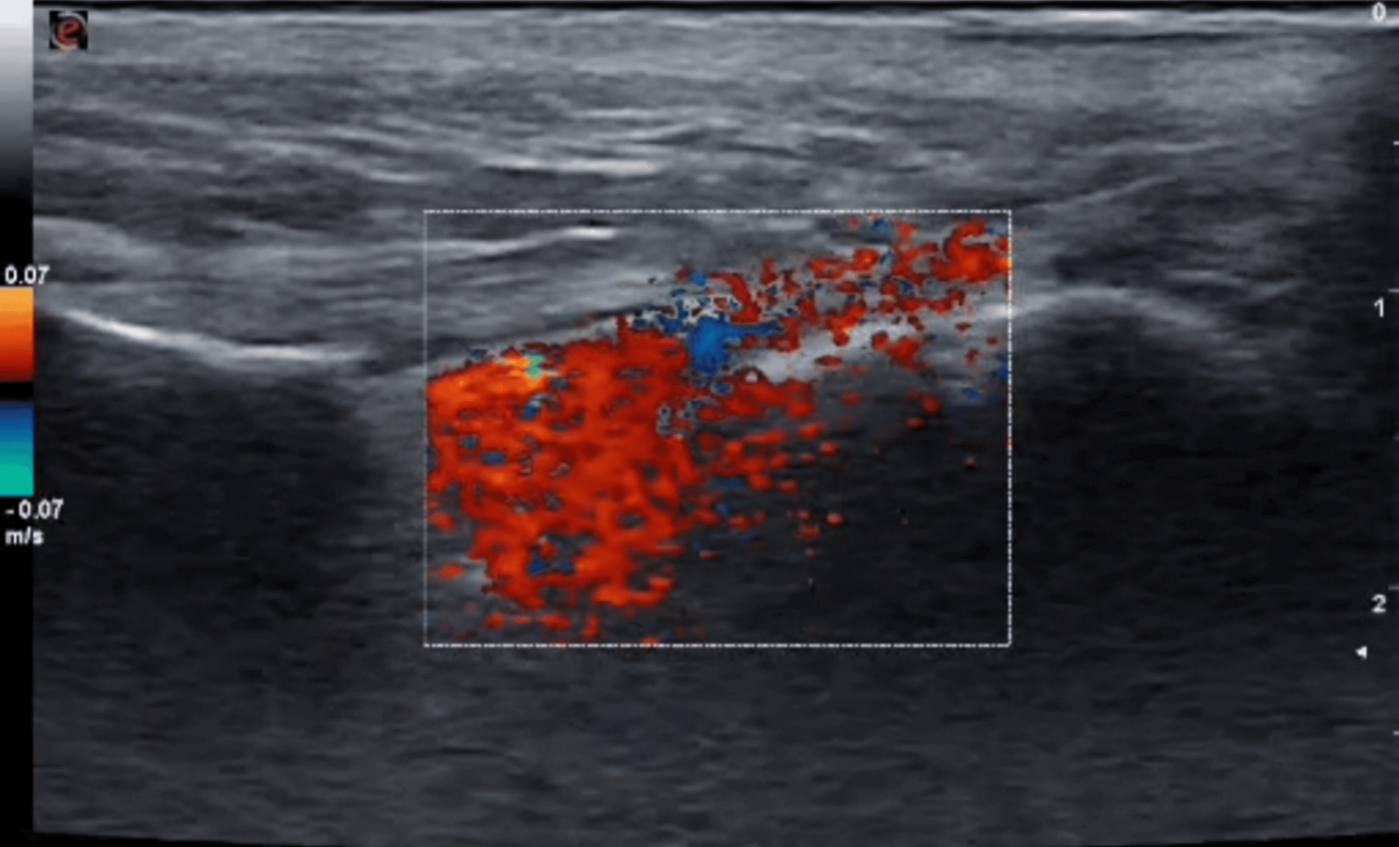
Color Doppler showing an appropriate flow of the local anesthetic

Needle placement was confirmed by real-time visualization of local anesthetic spread, supplemented by color doppler imaging, and 15 mL of 0.325% bupivacaine was administered (Figure 3) after negative aspiration to achieve a good amount of motor block and to facilitate a comfortable lithotomy position for the surgery.

Loss of resistance technique was not routinely employed, as ultrasound visualization was considered sufficient for accurate placement. All blocks were performed by experienced anesthesiologists familiar with USG regional techniques to ensure procedural consistency.

Group S: Saddle Block

Thirty patients were allotted to this group. Saddle block was performed in the sitting position using a 25G Quincke spinal needle (Becton, Dickinson and Company (BD), Franklin Lakes, NJ, USA) at the L4-L5 interspace under standard aseptic precautions. After confirmation of cerebrospinal fluid free flow, 2 mL of 0.5% hyperbaric bupivacaine was injected intrathecally, and patients were maintained in the sitting position for 10 minutes. After achievement of an adequate level of anesthesia, the patient was repositioned to the lithotomy position for the surgery.

Sensory block was assessed using pinprick with a 22G hypodermic needle over sacral dermatomes (S2-S5) at two-minute intervals until adequate block was achieved.

Postoperative monitoring

Hemodynamic parameters were recorded at specific intervals. Postoperative pain was measured using the Visual Analog Scale (VAS). Rescue analgesia was administered when the Visual Analog Scale (VAS) score was ≥four using intravenous tramadol 50mg, with repeat dosing permitted every eight hours as required. The time to first rescue analgesia (VAS ≥4), time to mobilization, time to voiding, patient and surgeon satisfaction scores, and adverse events were all documented.

Outcome measures

The primary outcome measure was time to mobilization, defined as the duration (in hours) from completion of the block to the patient’s ability to stand and ambulate without assistance, with no evidence of gait instability, dizziness, or orthostatic hypotension. Secondary outcomes included onset time of sensory block assessed by the pinprick method, degree of motor blockade evaluated using the Modified Bromage scale [10], intraoperative hemodynamic changes (heart rate, systolic, diastolic, and mean arterial pressure), time to first rescue analgesia (VAS ≥4), and time to first voiding. Additional parameters recorded were requirement of supplemental local anesthetic infiltration, patient and surgeon satisfaction scores (four-point Likert scale), and incidence of adverse events.

Statistical analysis

Statistical analysis was performed using IBM SPSS Statistics for Windows, Version 25 (Released 2017; IBM Corp., Armonk, New York, United States). Continuous variables were expressed as mean ± standard deviation and compared using independent Student’s t-test or Mann-Whitney U test as appropriate. Categorical variables were analyzed using Chi-square or Fisher’s exact test. The primary outcomes (time to mobilization and time to first rescue analgesia) and key secondary outcomes were further analyzed by calculating effect sizes (Cohen’s d for continuous variables) along with 95% confidence intervals to provide a measure of the magnitude and precision of observed differences. This was done to complement p-values and allow better interpretation of clinical relevance. All statistical tests were two-tailed, and a p-value of <0.05 was considered statistically significant.

## Results

Demographic and baseline characteristics

The two groups were statistically comparable with respect to age, BMI, ASA physical status, and duration of surgery (all p >0.05) (Table 1).

**Table 1:** Demographic and baseline characteristics

Parameter	Group C (n = 29)	Group S (n = 30)	p value
Age (years)	41.2 ± 8.4	39.8 ± 7.9	0.42
BMI (kg/m²)	24.8 ± 3.1	25.1 ± 2.9	0.61
ASA			0.89
Grade I	15	16	
Grade II	11	10	
Grade III	3	4	
Duration of surgery (min)	32.4 ± 6.8	31.7 ± 7.2	0.67

This indicates successful randomization and homogeneity between groups at baseline. The similarity in surgical duration also reduces procedural bias that can impact changes in blood flow during surgery or recovery after the operation. Since there were no major demographic differences, any subsequent variations in pain relief duration, mobilization time, and blood flow stability can be fairly linked to the anesthetic technique instead of other influencing factors. 

Continuous variables were analyzed using independent t-tests (df=57), while categorical variables were assessed using the Chi-square test (df=2). The effect sizes were small to negligible (Cohen’s d ranging from 0.10 to 0.17; Cramér’s V=0.06), indicating that the two groups were well matched at baseline and that randomization was effective.

Intraoperative and post-operative parameters

Saddle block had a much quicker sensory onset than USG-CEB (p<0.001). This shows the fast effect of the intrathecal local anesthetic. However, the postoperative recovery results were better for the caudal group. They experienced significantly earlier mobilization (p<0.001), earlier voiding (p<0.001), and a longer time before needing the first rescue analgesia (p=0.021; Table 2).

**Table 2:** Intraoperative and post-operative parameters CI: confidence interval; d: Cohen’s effect size.

Outcome	Group C	Group S	Mean difference (95% CI)	Cohen’s d	p-value
Procedure time (min)	5.41 ± 0.92	4.43 ± 0.80	0.98 (0.55 to 1.41)	1.13	0.018
Sensory onset (min)	12.86 ± 1.84	9.83 ± 1.42	3.03 (2.20 to 3.86)	1.84	<0.001
Time to first analgesia (h)	5.00 ± 0.95	4.45 ± 0.74	0.55 (0.12 to 0.98)	0.64	0.021
Mobilization time (h)	5.82 ± 0.71	6.95 ± 0.60	-1.13 (−1.47 to −0.79)	1.73	<0.001
Time to voiding (h)	4.95 ± 0.73	6.82 ± 0.71	-1.87 (−2.24 to −1.50)	2.6	<0.001

Although procedure time was marginally longer in the caudal group (p=0.018), the improved recovery profile suggests that USG-CEB may be more suitable for ambulatory anorectal surgeries where early discharge is desirable. Independent t-test analysis (df=57) showed significant differences between the groups for key perioperative outcomes. The effect sizes were moderate to very large, with Cohen’s d values of 1.13 for procedure time, 1.86 for sensory onset, 1.73 for mobilization time, and 2.60 for time to voiding, while time to first analgesia showed a moderate effect (d=0.64). These findings suggest that the observed differences are not only statistically significant but also clinically meaningful.

Hemodynamic parameters

Baseline hemodynamic parameters were statistically comparable between groups. The saddle block group demonstrated significantly greater reductions in heart rate at five and 10 minutes and in systolic, diastolic, and mean arterial pressures at all early intraoperative intervals (all p<0.05; Table 3).

**Table 3:** Comparison of hemodynamic parameters between groups Group C: Ultrasound-guided caudal epidural block; Group S: saddle block.

Parameter	Time	Group C (n=29)	Group S (n=30)	Mean difference (95% CI)	p-value
Heart rate (bpm)	Baseline	78.6 ± 8.1	77.9 ± 7.8	0.7 (−3.5 to 4.9)	0.74
	5 min	76.8 ± 7.6	73.2 ± 7.9	3.6 (−0.5 to 7.7)	0.082
	10 min	77.2 ± 7.4	71.8 ± 7.6	5.4 (1.1 to 9.7)	0.018
	15 min	78.1 ± 7.2	72.4 ± 7.8	5.7 (1.4 to 10.0)	0.015
	20 min	77.6 ± 7.0	72.1 ± 7.5	5.5 (1.3 to 9.7)	0.017
Systolic blood pressure (mmHg)	Baseline	124.2 ± 10.1	123.5 ± 9.8	0.7 (−4.6 to 6.0)	0.78
	5 min	121.8 ± 9.7	114.3 ± 10.5	7.5 (1.8 to 13.2)	0.011
	10 min	120.6 ± 9.3	110.4 ± 10.8	10.2 (4.5 to 15.9)	0.003
	15 min	119.8 ± 9.1	109.7 ± 10.2	10.1 (4.6 to 15.6)	0.004
	20 min	120.2 ± 8.9	110.1 ± 9.8	10.1 (4.8 to 15.4)	0.003
Diastolic blood pressure (mmHg)	Baseline	78.4 ± 7.2	77.6 ± 7.0	0.8 (−2.9 to 4.5)	0.67
	5 min	76.2 ± 6.5	71.4 ± 7.1	4.8 (1.0 to 8.6)	0.014
	10 min	75.1 ± 6.1	69.3 ± 7.0	5.8 (2.0 to 9.6)	0.008
	15 min	74.6 ± 6.0	68.7 ± 6.8	5.9 (2.3 to 9.5)	0.009
	20 min	75.0 ± 5.8	68.9 ± 6.6	6.1 (2.5 to 9.7)	0.007
Mean arterial pressure (mmHg)	Baseline	93.7 ± 8.1	92.9 ± 7.8	0.8 (−3.5 to 5.1)	0.71
	5 min	89.5 ± 7.2	83.7 ± 7.8	5.8 (1.5 to 10.1)	0.012
	10 min	87.8 ± 6.4	80.9 ± 6.7	6.9 (2.8 to 11.0)	0.021
	15 min	87.1 ± 6.3	80.3 ± 6.5	6.8 (2.9 to 10.7)	0.019
	20 min	87.4 ± 6.1	80.6 ± 6.4	6.8 (3.1 to 10.5)	0.018

Patients in the caudal group, on the other hand, consistently demonstrated higher heart rate and blood pressure values, with statistically significant differences at multiple time points. The confidence intervals for these differences did not cross zero, supporting the robustness of these findings. Overall, these results indicate better hemodynamic stability with CEB, likely due to its more localized sympathetic blockade compared to saddle block.

Requirement of local infiltration

Supplemental local anesthetic infiltration was required significantly more often in the caudal group (p=0.004; Table 4).

**Table 4:** Requirement of local infiltration Group C: Ultrasound-guided caudal epidural block; Group S: saddle block.

Parameter	Group C	Group S	p value
Required	7 (24.1%)	0 (0%)	0.004
Not required	22 (75.9%)	30 (100%)	

This may reflect incomplete sacral spread or variability in epidural distribution despite ultrasound guidance. However, the majority (75.9%) of patients in the caudal group achieved adequate surgical anesthesia without supplementation. Although the saddle block provided more uniformly dense anesthesia, the additional infiltration in selected caudal cases did not adversely affect overall satisfaction or recovery outcomes. The requirement for supplemental local infiltration was analyzed using the Chi-square test (df=1). The effect size was moderate to large (Cramér’s V=0.32), indicating that the increased need for infiltration in the caudal group was not only statistically significant but also clinically relevant.

Satisfaction scores

Patient and surgeon satisfaction scores were high and comparable between the two groups (p>0.05; Table 5).

**Table 5:** Satisfaction scores (four-point Likert scale) Group C: Ultrasound-guided caudal epidural block; Group S: saddle block.

Outcome	Group C	Group S	Risk Ratio (95% CI)	p-value
Surgeon satisfaction	27 (93%)	28 (93%)	1.00 (0.85–1.17)	0.97
Patient satisfaction	26 (90%)	25 (83%)	1.08 (0.89–1.31)	0.41

Despite slower sensory onset and occasional need for supplemental infiltration in the caudal group, overall surgical conditions were satisfactory. Similarly, although saddle block was associated with greater hemodynamic fluctuations and delayed mobilization, patient-perceived comfort remained acceptable. Satisfaction scores were compared using the Chi-square test (df=1). The effect sizes were small to negligible (Cramér’s V ranging from 0.01 to 0.12), suggesting that patient and surgeon satisfaction were comparable between the two groups. However, the wide confidence intervals suggest limited precision, and given the study was not powered for this outcome, these findings should be interpreted cautiously.

## Discussion

In this study, USG-CEB demonstrated several advantages over the saddle block in terms of postoperative recovery, while maintaining comparable intraoperative anesthetic efficacy. Patients in the caudal group experienced earlier mobilization, earlier return of bladder function, and more stable hemodynamic profiles. In contrast, the saddle block was associated with a faster onset of sensory blockade. These findings are consistent with prior comparative studies evaluating caudal and spinal anesthesia techniques in anorectal surgery [11,12].

The improved recovery characteristics observed with CEB can be explained by its more localized mechanism of action. Unlike spinal anesthesia, which produces a relatively extensive sympathetic blockade, caudal epidural anesthesia primarily affects the sacral nerve roots, thereby preserving hemodynamic stability and facilitating earlier mobilization [13]. This advantage is particularly relevant in ambulatory surgical settings, where early recovery plays a key role in discharge planning and patient throughput. In addition, the earlier return of bladder function observed in the caudal group aligns with the known association between spinal anesthesia and postoperative urinary retention [14].

The role of USG further strengthens the applicability of the caudal technique. Anatomical and technical studies have demonstrated that visualization of the sacral hiatus and surrounding structures improves the accuracy of needle placement and reduces failure rates [15]. Earlier work has also highlighted the challenges associated with blind caudal epidural placement, including variability in anatomical landmarks and a higher likelihood of incorrect needle positioning [16]. By overcoming these limitations, USG enhances both the reliability and safety of CEB in adult patients.

Despite these advantages, certain trade-offs must be acknowledged. In the present study, the onset of sensory blockade was significantly slower in the caudal group compared to saddle block. This finding is consistent with previous reports comparing neuraxial techniques, where spinal anesthesia typically provides a more rapid onset due to direct intrathecal drug delivery [17]. In addition, a subset of patients in the caudal group required supplemental local infiltration to achieve adequate surgical anesthesia. This suggests that while caudal block is effective, it may not consistently provide complete surgical conditions in all patients, particularly when compared to the more predictable spread of intrathecal anesthesia [18].

The duration of postoperative analgesia was modestly longer in the caudal group. Although statistically significant, the absolute difference was relatively small, and its clinical relevance may vary depending on the use of multimodal analgesic strategies. Previous studies evaluating regional anesthesia techniques in anorectal surgery have similarly reported variability in analgesic duration, emphasizing the importance of individualized pain management approaches [19].

Hemodynamic stability emerged as a consistent advantage of the CEB in this study. Patients in the caudal group maintained higher heart rate and blood pressure values intraoperatively, reflecting reduced sympathetic blockade. These findings are supported by broader evidence in regional anesthesia, which suggest that techniques with more limited sympathetic involvement are associated with fewer hemodynamic fluctuations [20]. In clinical practice, this may reduce the need for vasopressor support and contribute to a smoother intraoperative course.

Patient and surgeon satisfaction were comparable between the two groups. While this indicates that both techniques are acceptable in routine practice, it is important to note that the study was not powered to detect differences in satisfaction outcomes. Furthermore, satisfaction is a multifactorial outcome influenced by perioperative experience, expectations, and overall recovery, rather than anesthetic technique alone [21].

Several limitations of this study should be considered when interpreting the findings. The sample size was relatively small, and the study was conducted at a single center, which may limit generalizability. The performance of USG-CEB is inherently operator-dependent, and outcomes may vary based on experience and expertise. Additionally, different concentrations of local anesthetic were used in the two groups, which may act as a confounding factor and limit direct comparison of the techniques themselves. Blinding of patients and the performing anesthetists was not feasible due to differences in the positioning and procedural characteristics, which may introduce bias.

Another limitation is the focus on immediate perioperative outcomes, without assessment of longer-term recovery parameters. Future studies incorporating extended follow-up and patient-reported outcome measures would provide a more comprehensive understanding of the clinical impact of these techniques. Comparative studies specifically evaluating USG versus landmark-based caudal approaches would also help clarify the independent contribution of USG.

Finally, it is important to consider these findings within the broader context of perioperative care. Neuraxial anesthesia techniques have been associated with improved postoperative outcomes, including reduced morbidity and enhanced recovery in various surgical populations [22]. In this context, the present study adds to the growing body of evidence supporting the role of targeted regional anesthesia techniques in optimizing recovery in ambulatory surgery.

## Conclusions

USG-CEB appears to be a practical and effective alternative to the saddle block for anorectal surgery, offering a favorable recovery profile with earlier mobilization, earlier return of bladder function, and greater hemodynamic stability. At the same time, these advantages should be interpreted alongside certain limitations, including a slower onset of anesthesia and the need for supplemental local infiltration in a subset of patients. Although the differences in recovery parameters were statistically significant, their clinical impact may vary depending on institutional practices and discharge protocols. Overall, both techniques provide reliable anesthesia, and the choice between them should be individualized, based on patient characteristics, surgical requirements, and clinician expertise. Further, larger multicenter studies with extended follow-up are warranted to better define the role of USG-CEB in adult ambulatory anorectal surgery.
